# Features of hypersensitivity to local anesthetics based on articainum in tests in vitro

**DOI:** 10.1186/2045-7022-4-S3-P130

**Published:** 2014-07-18

**Authors:** Tair Nurpeissov, Temyrlan Nurpeissov, Aizhan Avdugalieva

**Affiliations:** 1 SRI of Cardiology and Internal Diseases, Kazakhstan; 2 SRI of Cardiology and Internal Diseases, Republican Allergological Center, Kazakhstan

## Background

The considerable height of drug allergy is presently marked and local anesthetics are one of its frequent causes. Lately Articainum and his analogues is most widely used. Articainum is local anesthetic of group of amides, having instead of benzol circle of tiophene and additional aethereal group. In medicine composition auxiliary substances including epinephrine are used. Allergic reactions to it meet less often than to Novocain, however they are possible. There are several analogues of articainum on the market.

## The aim

To define the features of hypersensitivity on local anesthetics based on Articainum of different firms-producers.

## Method

The investigation exposed to 492 patients. These patients with complicated allergic anamnesis were sent by dentists to realization of allergy tests in vitro for few preparations including a few analogues of Articainum. Age of patients was 2 -- 81 yrs, averaging 25.0 ± 1.6. yrs. (61.2% women). The representatives of articainum (Ubistezinum®, Ubistezinum forte®, Articainum®, Ultracainum®) were used. In dough in vitro was put 1035 tests (not counting controls), up to 4 for a patient, on the average there was 2.10 tests for a patient. 447 tests appeared positive result (43.2%). Drug cytotoxic test (the Rate of neutrophils' damage) was used.

## Results

At testing of Articainum® it was determined 16 tests positive from 247 (6.5 ± 2.2%), for Ultracainum® -- 13/227 (5.7 ± 2.1%). Ubistezinum® and Ubistezinum® forte 70/332 (21.1 ± 3.6%) and from 61/229 (20.4 + 3.6%), respectively. Thus, four basic representatives of local anesthetics gave different indexes, divided into 2 groups - with the low and moderate level of sensitization. The educed distinctions between groups were reliable (ð<0.01). For further treatment "negative" local anestetics were applicated. No allergic reactions were registered.

## Conclusion

1. Drug cytotoxic test showed well applicability for prevention of drug hypersensitivity for local anesthetics. 2. Thus, local anesthetics based on articainum and produced by different providers have shown considerable difference on the profile of safety concerning drug allergy. There is an evidence of greater safety of Articainum® and Ultracainum®. 3. It confirms a thesis about dependence of drug allergy not only on a basic substance but also from other descriptions -- concentration, type of auxiliary substances, etc. 4. The importance of the testing of exactly that preparation that is planned to the further use is proven.

